# Renal Status in Newly Diagnosed Patients with Diabetes Mellitus: A Descriptive Study in Primary Care and Opportunities for Improving Management

**DOI:** 10.3390/jcm14082732

**Published:** 2025-04-16

**Authors:** Pilar Vich-Pérez, Belén Taulero-Escalera, Paula Regueiro-Toribio, Almudena Cárdenas-de Miguel, Rebeca San Román Muñoz, Miguel A. Salinero-Fort, on behalf of the LADA-PC Consortium

**Affiliations:** 1 Hospital La Paz Institute for Health Research (IdiPAZ), 28029 Madrid, Spain; 2 Los Alpes Health Centre, 28022 Madrid, Spain; almudena.cardenas@salud.madrid.org (A.C.-d.M.);; 3 Foundation for Biosanitary Research and Innovation in Primary Care, 28003 Madrid, Spain; belentauesc@gmail.com; 4 Mar Báltico Health Centre, 28033 Madrid, Spain; paula.regueiro@salud.madrid.org

**Keywords:** diabetes mellitus, chronic kidney disease, albuminuria, risk factors, cross-sectional studies

## Abstract

**Background/Objectives:** The current study aims to estimate the frequency of abnormal renal status (ARS, defined as chronic kidney disease (CKD) diagnosis in electronic medical records or current albuminuria) in people with newly diagnosed diabetes mellitus (DM), to determine the associated risk factors, and to evaluate the level of compliance with good clinical practice recommendations. **Methods:** Cross-sectional study with 1030 adults diagnosed with DM in the last 4 years. Anthropometric, clinical, analytical, and lifestyle variables were collected. Multivariate analyses were performed to determine the factors associated with ARS. **Results:** Hypercholesterolaemia, metabolic syndrome, hypertension, obesity, hypertriglyceridaemia, and cardiovascular disease (CVD) were the most prevalent comorbidities. ARS was present in 11.5% of patients. The variables associated with ARS were male sex (OR: 1.78; 95% CI, 1.16–2.75), age ≥70 years (OR: 2.96; 95% CI: 1.92–4.56), hypertension (OR: 1.59; 95% CI: 1.03–2.44), CVD (OR: 1.73; 95% CI: 1.03–2.90), and hemoglobin A1c (HbA1c) ≥8% (OR: 2.26; 95% CI, 1.19–4.27). Among patients with hypertension and albuminuria, 80% received angiotensin-converting enzyme inhibitors (ACE inhibitor) or an angiotensin receptor blocker (ARB), compared to 60% of those with albuminuria without hypertension. The 42.4% patients with ARS were treated with sodium-glucose cotransporter type 2 inhibitors (SGLT2i) and 72% with statins, but only 31.5% achieved the target low density lipoproteins cholesterol (LDLc) < 70 mg/dL. **Conclusions:** ARS in newly diagnosed patients with DM is less common than described in the literature, but risk factors for its development are highly prevalent. Adherence to good clinical practice recommendations was poor, especially in LDL cholesterol targets and the use of SGLT2i.

## 1. Introduction

Chronic kidney disease (CKD) is defined as the presence of abnormalities in kidney structure or function that have been present for at least 3 months and manifests as a glomerular filtration rate of less than 60 mL/min/1.73 m^2^ and/or markers of kidney damage. Among them, the most common is albuminuria greater than 30 mg/g [1,2,3]. CKD is classified according to cause, glomerular filtration rate (G1--G5), and albuminuria category (A1–A3) [3].

CKD is a significant public health problem worldwide, with an estimated prevalence of 9–13% [4,5], although it is underdiagnosed [6].

The most common causes of CKD are diabetes mellitus (DM) and arterial hypertension (HT). However, many other risk factors favor its development, among which the following stand out: advanced age, certain ethnic groups, low socioeconomic status, family history of CKD, congenital renal pathologies, cardiovascular disease (CVD), dyslipidemia, obesity, metabolic syndrome, smoking, alcoholism, glomerulopathies, interstitial kidney disease, severe or recurrent urinary tract infections, drugs such as nonsteroidal anti-inflammatory drugs, and some antibiotics [3].

People with CKD have a significantly increased cardiovascular risk [7,8], and their risk of mortality is increased by 5–10 times [2,5].

DM is a significant risk factor for the development of CKD in developed countries. It is estimated that up to 40% of people with DM may develop CKD during their disease [2], which may be present at diagnosis in the case of type 2 diabetes mellitus (T2DM) and after 5–10 years of evolution in type 1 diabetes mellitus (T1DM). However, CKD can be prevented by appropriate lifestyle choices, the avoidance of nephrotoxic drugs, and the control of cardiovascular risk factors and blood glucose levels [2,3].

In addition to these measures, once CKD is established, its progression can be slowed by the use of certain drugs, such as angiotensin-converting enzyme inhibitors, angiotensin II receptor antagonists (ACEi/ARBs), sodium-glucose cotransporter type 2 inhibitors (SGLT2i), and mineralocorticoid receptor antagonists, which, with their specific indications, are helpful in multiple studies and are recommended in good clinical practice guidelines [2,3].

Abnormal renal status (ARS) is a proxy for kidney damage that can be easily used in primary care if it is based on previously diagnosed CKD in electronic medical records (EMRs) or current albuminuria.

Few studies investigated the renal status of patients with DM in the early stages of the disease. Therefore, the present study aims to determine the current situation of ARS in primary care patients with newly diagnosed DM in Madrid (Spain), the associated risk factors, and the use of medications according to clinical practice recommendations. This will allow us to increase therapeutic efforts in those patients who need it most, since prevention and control measures have the greatest benefit in the early stages of DM.

## 2. Materials and Methods

This is an observational, descriptive, multicenter, cross-sectional study carried out in a primary care setting in the Community of Madrid (Spain) that includes 1030 patients with diabetes that met the following criteria: over 30 years of age, diagnosed in the last 4 years, and with at least 6 months of evolution.

The exclusion criteria included: gestational diabetes, being homebound, and having a severe degenerative neurological or psychiatric disease.

The study was approved by the regional ethics committee (Code: JCAH/PVP/LADA/2019/1. Version: 1 of 4 March 2019).

One hundred eighty-four researchers, as well as medical and nursing professionals from 12 health centers attached to the same referral hospital, participated. This study is part of a larger study with a published protocol [9].

Once patients agreed to participate in the study and signed the informed consent form, blood and urine samples were taken. Within one to four weeks of the first visit, patients were re-assessed to complete the rest of the study variables and were informed of the results of the analyses.

The data sources included clinical interviews, EMRs, physical examinations, and analytical tests (blood count, biochemistry, urine, and albumin-creatinine ratio).

### 2.1. Variables

Sex: male, female.

Age: age at the time of DM diagnosis and at the time of study entry.

Renal status was determined via two criteria: (1) the diagnosis of CKD in the EMRs, which met the criteria of clinical practice guidelines [3] on the basis of medical judgement, and (2) the measurement of albuminuria by the most recent albumin–creatinine ratio (ACR). The absence of a CKD diagnosis in the EMRs and the absence of albuminuria indicate normal renal status. In contrast, ARS is characterized by either a diagnosis of CKD in the EMRs or current albuminuria of 30 mg or more.

Hypercholesterolemia: if the diagnosis was recorded in the EMRs or if the patient had total cholesterol levels greater than 200 mg/dL on at least two occasions prior to treatment with lipid-lowering drugs.

Hypertension (HT): if the diagnosis was recorded in the EMRs or if the diagnosis was made by the patient’s healthcare professional according to current criteria.

Metabolic syndrome was considered when the requirements of the National Cholesterol Education Program Adult Treatment Panel III were met: at least three of these components: (1) abdominal circumference ≥102 cm in men and ≥88 cm in women; (2) triglycerides ≥150 mg/dL; (3) blood pressure ≥130/85 mm/Hg or established hypertension; (4) high density lipoproteins cholesterol (HDLc) <40 mg/dL in men and <50 mg/dL in women; and (5) fasting plasma glucose between 110 and 126 mg/dL (6.11 to 6.99 mmol/L) or a diagnosis of DM [10,11].

Hypertriglyceridaemia: if the diagnosis was recorded in the EMRs or if the diagnosis was made by the referring health professional on the basis of current criteria.

Cardiovascular disease (CVD): Presence of ischaemic heart disease, stroke, or peripheral artery disease as recorded in the EMRs. These variables have been validated previously in EMRs [12].

Obesity was defined as a body mass index (BMI) equal to or greater than 30 kg/m^2^ at the time of inclusion.

Physical activity level: according to the International Physical Activity Questionnaire (IPAQ), the defined categories are reduced version [13] low or sedentary (no physical activity, or activity performed is not sufficient to reach IPAQ category 2 or 3), moderate (≥3 sessions per week of vigorous physical activity for at least 20 min per day; or ≥5 sessions per week of moderate physical activity and/or walking for at least 30 min per day; or ≥5 sessions per week of any combination of walking and/or moderate and/or vigorous physical activity), and high physical activity (≥3 sessions per week of vigorous physical activity for at least 60 min per day, or ≥7 sessions per week of any combination of walking and/or moderate and/or vigorous intensity physical activity).

Regarding smoking, three categories were established: active smoker (currently smokes, regularly or occasionally), non-smoker (never smoked), and ex-smoker (quit smoking and has not smoked since at least one year).

Consumption of alcohol: Units of alcohol consumed were measured every week. One unit (10 g of alcohol) is equivalent to a glass of wine (100 cc), a glass of beer (200 cc), half a glass of vermouth (50 cc), or half a glass of whiskey or cognac (25 cc). The quantities were measured as follows: small glass, 125 mL; medium glass, 200 mL; large glass, 250 mL; glass/mixed drink, 50 mL; bottle of beer, 200 mL; and bottle of wine, 750 mL. After recording the amounts of alcohol in the electronic notebook, a calculator integrated into the system measured daily and weekly alcohol ingestion, classifying patients into risk drinkers (>40 g of alcohol/day for men, >24 g for women), non-risk drinkers (>0 and <40 g of alcohol/day for men, >0 and <24 g for women), and non-drinkers.

Mediterranean diet: A modified version of the Mediterranean Diet Adherence Screener (MEDAS) questionnaire was used to assess adherence to the Mediterranean diet (11–14 points indicated high adherence, 6–10 points implied medium adherence, and a score of 0–5 meant low adherence). The modification concerns the item on wine consumption, taking into account two drinks per week instead of seven, as in the original MEDAS, to screen for alcohol consumption, as a variable was available that was categorized by risk on the basis of the type of drink and grams/week, not just the number of drinks. This questionnaire might be useful in clinical practice and has been shown to be a valid tool for rapid assessment of adherence and may be useful in clinical practice [14].

Polypharmacy: patients were considered to be polymedicated when they consumed five or more drugs per day, as reported in different studies [15].

Treatment with ACE inhibitors or ARBs: treatment with the following drugs was recorded in the EMRs: benazepril, captopril, enalapril, fosinopril, lisinopril, perindopril, quinapril, ramipril, trrandolapril, candesartan, eprosartan, irbesartan, losartan, olmesartan, telemisartan, or valsartan.

Treatment with SGLT2i: treatment with the following drugs was included in the EMRs: canagliflozin, empagliflozin, dapagliflozin, or ertugliflozin.

Treatment with lipid-lowering agents: treatment with the following drugs was included in the EMRs: atorvastatin, pitavastatin, pravastatin, rosuvastatin, lovastatin, simvastatin, ezetimibe, gemfibrozil, and fenofibrate.

LDL cholesterol (LDLc) levels were <70 mg/dL or <100 mg/dL if the patient had LDLc levels <70 mg/dL or <100 mg/dL, respectively, in the previous 6 months.

### 2.2. Statistical Analysis

Descriptive analysis was performed for all variables included in the study, stratified by sex and age. Continuous quantitative variables are expressed as the means and standard deviations or medians and interquartile ranges, where appropriate, depending on the type of distribution. Qualitative variables were summarized as relative and absolute frequencies (%).

Student’s *t* test or the nonparametric Mann-Whitney U test for nonnormally distributed data were used to compare subgroups of quantitative variables. The chi-square test or z test for comparing proportions was used to compare qualitative variables.

Univariate analysis was performed to determine the odds ratio (OR) of the ARS for each variable, followed by multivariate analysis via binary logistic regression to determine the variables significantly associated with the ARS. Variables were entered into the model stepwise according to statistical significance in univariate analysis (*p* < 0.05), and those variables were considered relevant according to previous findings. We used the backwards LR method (entry: 0.05, removal: 0.10). The final model was tested for goodness of fit via the Hosmer–Lemeshow test, which assumes normality of the residuals and homoscedasticity of the variance.

## 3. Results

The sample comprised 56.1% men with a mean age of 62.76 years (SD: 10.81). Women were older (64.56 (SD:11.32) vs. 61.35 (SD: 10.18) men, *p* < 0.001). The mean age at onset of DM was 59.57 (SD: 10.70), with men being younger (58.15 (SD: 10.00) vs. 61.38 women (SD: 11.28), *p* < 0.001) (Table 1).

In our study, 118 patients (11.5%) were classified as having ARS, and 8.7% had albuminuria. There were sex differences in ARS (13.3% in men vs. 9.1% in women, *p* = 0.034) and albuminuria (10.5% in men vs. 6.7% in women, *p* < 0.01). Among women, 4.5% had ARS under the age of 60, whereas 12% were over the age of 60 (*p* < 0.01). Among men, 10.5% of those over 60 years of age had ARS, whereas 16.1% of those over 60 years of age had ARS (*p* = 0.047).

The most common comorbidities in newly diagnosed patients with DM were hypercholesterolaemia (64.7%), metabolic syndrome (58.7%), HT (55.7%), obesity (49.4%), hypertriglyceridaemia (26.4%), and CVD (11.7%).

Men were significantly more likely to have CVD and hypertriglyceridaemia. Conversely, women were more likely to utilize polypharmacy than men were (Table 1).

In both sexes, patients older than 60 years were more likely to have ARS, hypercholesterolaemia, hypertension, CVD, and polypharmacy than younger patients were.

Fifty-one percent of the patients studied had a sedentary attitude, being more common in women than in men (58.0% vs. 45.8%, *p* < 0.01), in women, those older than 60 years (63.5% vs. 49.4%; *p* < 0.01), and in men, those younger than 60 years (51.7% vs. 40.1%; *p* < 0.01).

In terms of alcohol consumption, 47.8% of the participants were drinkers, more frequently men (53.8% vs. 30.5%, *p* < 0.001), and within these, they were older than 60 years (59.4% vs. 48.0%, *p* < 0.01). Smoking accounted for 18.5% of the total, more frequently in men (21.6% vs. 14.5%, *p* < 0.01), and within these, in young men (25.6% vs. 17.7%, *p* = 0.021).

With respect to the Mediterranean diet, 7.4% of individuals had low adherence to the diet, with low adherence being more common among men (9.0% vs. 5.4%, *p* = 0.027) and among those under 60 years of age (12.5% vs. 5.6%, *p* < 0.01).

**Figure 1:**
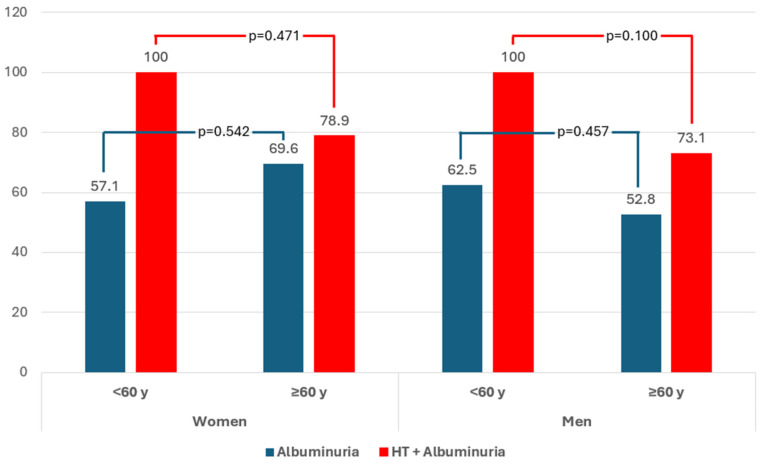
Proportion of the use of angiotensin-converting enzyme inhibitors or angiotensin receptor blockers in patients with newly diagnosed diabetes according to hypertension and albuminuria. Stratified by sex and age group. HT: hypertension.

Among patients with DM, HT and albuminuria, 80% were receiving ACEi/ARB therapy. There were no significant differences in the use of this therapy by age in either sex. Among patients who had albuminuria but not hypertension, 60% were on ACEi/ARB therapy, with no significant differences according to sex or age (Table 2 and Figure 1).

**Figure 2:**
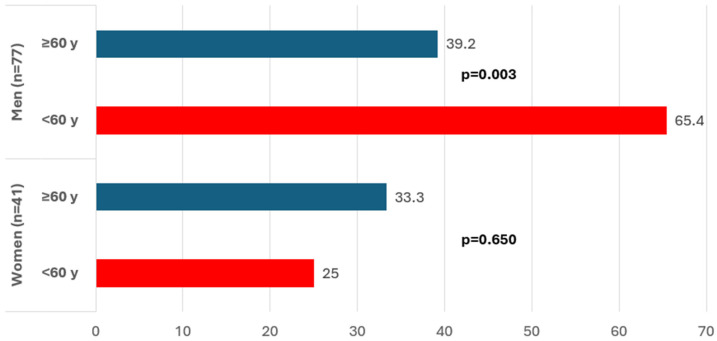
Proportion of the use of SGLT2is in patients with newly diagnosed diabetes mellitus and ARS. Stratified by sex and age group; SGLT2i: sodium-glucose cotransporter type 2 inhibitors.

Among patients with ARS, 42.4% were treated with SGLT2i without differences by gender. Figure 2 shows that among men, the use of SGLT2is in patients with ARS was more common among younger patients (65.4% under the age of 60 years than among 39.2% over the age of 60 years, *p* = 0.03).

**Figure 3:**
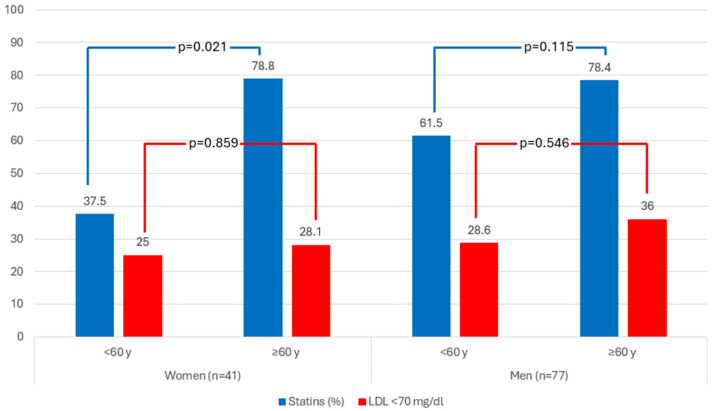
Statin use and optimal LDL cholesterol control (<70 mg/dL) in patients with newly diagnosed diabetes and ARS.

In terms of statin use, 72% of patients with ARS were on treatment, with differences by age within the women group (78.8% in the >60 years group vs. 37.5% in the <60 years group, *p* = 0.034) (Table 2, Figure 3).

Finally, according to the recommendations proposed by good clinical practice guidelines, which suggest maintaining LDL cholesterol < 70 mg/dL for patients with DM and at least one additional cardiovascular risk factor, only 31.5% of patients met this target, with no significant differences in compliance based on sex or age (Table 2, Figure 3).

Prior to conducting multivariate analysis, we examined the proportion of patients with abnormal renal status (ARS) stratified by social and clinical characteristics (Table 3). The multivariate logistic regression results reveal that the factors associated with ARS were being male (OR: 1.78, 95% CI, 1.16–2.75), being 70 years or older (OR: 2.96, 95% CI: 1.92–4.56), having hypertension (OR: 1.59, 95% CI: 1.03–2.44), having a history of CVD (OR: 1.73, 95% CI: 1.03–2.90), and having an HbA1c level ≥8% (OR: 2.26; 95% CI, 1.19–4.27) (Table 4).

## 4. Discussion

The present study, which was conducted in Madrid among patients recently diagnosed with DM, reported ARS of 11.5% and 8.7% for albuminuria.

It is estimated that up to 40% of people with DM will develop CKD during their lifetime. International studies suggest that the prevalence of CKD among people with DM is approximately 20% when diagnosis codes are used, 17.7% with an impaired estimated glomerular filtration rate (eGFR), 11.9% with albuminuria, and 32.7% when one or more methods that indicate CKD are used [16]. A CKD prevalence of 31.6% in newly diagnosed type 2 diabetes patients was reported in a United States study [17], and a similar study conducted in Mexico reported a CKD prevalence of 39.2% [18].

Various studies in Spain also analyzed CKD prevalence in people with DM. One of them reported a prevalence of approximately 34%, with a higher risk associated with male sex, as well as age and length of diabetes [19]. Additionally, the PREDIAB-CV study, a major research initiative, reported a CKD prevalence of 39.6% in people with DM and 17.7% in those with prediabetes [20].

However, the frequency of CKD observed in our study was significantly lower than that reported in these previous studies. This discrepancy may be attributed to our patients being diagnosed with diabetes at an earlier stage of progression and how this variable was measured. Furthermore, research conducted by the MADIABETES group in a cohort of T2DM patients coming from the same region as our sample documented a cumulative CKD incidence (period prevalence) of 10.23% after 5 years of follow-up [21]. The frequency of CKD in patients with T2DM in the MADIABETES study is similar to what we observed in our own research. However, it is important to note that the MADIABETES study included patients at various stages of disease progression, whereas our study focused solely on those with a maximum of four years of disease progression.

In relation to disease management, specifically regarding medications, the American Diabetes Association (ADA) and the Kidney Disease: Improving Global Outcomes (KDIGO) guidelines [22] recommend the use of ACE inhibitors or angiotensin receptor blockers for patients with DM who also have comorbid hypertension and microalbuminuria. In our study, 80% of patients were treated with these drugs, which is considered acceptable. Another guideline recommendation is the use of this class of drugs in people with DM and microalbuminuria. In our study, the proportion of patients meeting this recommendation was only 60%.

The KDIGO guidelines recommend the use of SGLT2i in patients with DM and CKD with renal filtration rates higher than 20 or 30 mL/min/1.73 m^2^, depending on the specific drug. In our study, only 42.4% of patients meeting these criteria were prescribed SGLT2i. Although we did not directly measure the glomerular filtration rate, we recorded filtration rates below 60 mL/min/1.73m^2^ and instances of albuminuria. A more significant percentage of younger men than older men were treated with SGLT2i, but this trend was not observed in women. This finding is contradictory, as studies have shown that SGLT2i maintain their benefits in advanced ages, although they should be adapted to the individual characteristics of each patient [23,24,25]. We believe that the low percentage of patients on SGLT2i treatment could be attributed to the lack of awareness of healthcare professionals during the study or to the existence of patients with filtration rates below 20 mL/min, where no new treatments were initiated.

Clinical practice guidelines recommend the use of lipid-lowering drugs for patients with DM and CKD due to their elevated cardiovascular risk. These patients should be treated with statins, and the target LDL cholesterol is set at a minimum of 70 mg/dL. In our study, 72% of participants with ARS and DM received lipid-lowering treatment. However, only 31.5% of the patients managed to achieve the target LDL cholesterol level of less than 70 mg/dL. Younger women faced particular challenges regarding access to lipid-lowering treatments; only 37.5% of those under the age of 60 years were receiving treatment, whereas 78.8% of those over 60 years were receiving treatment (*p* = 0.021). Additionally, when we considered a less stringent LDL target of less than 100 mg/dL, 62.2% of the participants reached that goal.

Clinical practice guidelines highlight the importance of early detection of CKD and appropriate management once a diagnosis is established [1,2,3]. However, few studies assessed CKD in the early stages of DM progression. Identifying CKD at this stage is crucial, as it enables the implementation of preventive measures and management strategies to improve long-term outcomes [26,27].

Our study revealed that while the frequency of ARS in newly diagnosed DM patients is not as high as that reported in other studies, these patients still present significant cardiovascular risk factors and comorbidities, such as hypercholesterolemia, metabolic syndrome, hypertension, CVD, and obesity, in the early stages of DM. In particular, obesity and sedentary lifestyles were especially common among young men. These data are worrying since they could predict a high incidence of CKD in these patients in the near future. Fortunately, these risk factors can be addressed through lifestyle changes and by optimizing lipid-lowering treatments as well as SGLT2i use.

Another finding of this research is the high frequency (41.4%) of polymedicated patients, which is explained by the abundance of comorbidities in this population.

The results of the logistic regression analysis indicate that the factors most strongly associated with the presence of ARS in this study are being male, being 70 years of age or older, having hypertension, having a history of CVD, and having an HbA1c level of 8% or higher. Some factors are well documented in the literature, with numerous studies highlighting their significant roles in the development and progression of CKD. For example, hypertension is a well-established risk factor for CKD, contributing to renal damage over time [28,29]. Additionally, advanced age and poor glycemic control have been consistently linked to an increased prevalence of CKD [30,31].

The association between male sex and ARS found in our study is consistent with some studies [32,33] but differs from others [34,35], as it is not clear that diabetic kidney disease is more common in men than in women. As men tend to have more harmful habits related to alcohol and tobacco consumption than women, this situation may help to explain the association between male sex and ARS. However, this association was adjusted for the variables included in the initial full multivariate model, which consisted of variables with statistical significance (*p* <0.05) and with relevance from previous studies. In addition, to avoid confounding, the automated backward stepwise method was manually checked for variations in odds ratios (ORs) that might indicate the presence of confounding factors.

Several studies reported a significant association between CKD and cancer [36], and autoimmune diseases [37].

However, our study did not find these associations, which may be due to differences in study populations, methodologies, or residual confounding.

Our study found an inverse association between obesity and ARS in univariate analysis, which is consistent with the findings of several studies [38,39].

However, in the multivariate analysis, we did not identify obesity as a statistically significant factor associated with ARS. It is important to note that other variables, such as age, could confound the results observed in the univariate analysis. Most similar studies reported a direct association between obesity and chronic kidney disease (CKD) [40].

According to all these results, some recommendations could be suggested to the health professionals of the area. It would be very beneficial to request a chronic kidney disease screening in patients with diabetes in its earliest stages, staging according to the KDIGO Guidelines, and the appropriate use of SGLT2 inhibitors and other nephroprotective drugs when indicated. It is also worth considering the need for lipid-lowering therapy in most patients and the achievement of LDL cholesterol targets based on cardiovascular risk.

This study has several notable strengths, particularly the focus on individuals with early-stage DM, as there are few published studies on this population; the wide range of variables examined, which improves our understanding of this group; and the inclusion of a large, multicenter sample that is representative of newly diagnosed DM patients in the city of Madrid (Spain).

## 5. Limitations

Limitations of the study include the following: First, the cross-sectional design does not allow causality to be established between the factors studied and the ARS. Second, the involvement of many investigators could lead to errors in data collection.

Secondly, some specific variables that might influence the development of ARS, such as breed, patient self-medication (including non-steroidal anti-inflammatory drugs [NSAIDs]), specific uses of some drugs such as aminoglycoside antibiotics, some chemotherapy drugs, and radiocontrast agents were not studied. A few of these were included in broader groups such as general analgesic medication and oncology medication.

Finally, the method used to identify patients with ARS and/or microalbuminuria is challenging. For the ARS, the criterion for diagnosis was based on either EMR entry or professional judgment according to clinical practice guideline definitions. However, this approach is not strictly consistent with the current definition of CKD. Only the most recent measurement in the last six months was considered for microalbuminuria without confirming whether previous data were available for diagnostic validation in cases of elevated microalbuminuria.

## 6. Conclusions

In conclusion, the results of this study indicate that the frequency of ARS in patients with recently diagnosed DM in Madrid is lower than that reported in the literature. However, there is a high prevalence of risk factors, including obesity, a sedentary lifestyle, hypertension, dyslipidemia, smoking, and metabolic syndrome, among these patients. Moreover, there is an important underuse of drugs that are recommended in clinical practice guidelines for these patients, such as SGLT2i, as well as insufficient use of statins, especially in women under 60 years of age. Additionally, many patients fail to achieve therapeutic targets with these drugs.

Therefore, it is crucial to optimize lifestyle changes and control CKD risk factors in these patients. Increasing the use of drugs that have been shown to improve the prognosis is also essential, with particular attention given to women, who are often at a disadvantage in this regard.

## Figures and Tables

**Table 1:** Age, comorbidities, and lifestyle in individuals with newly diagnosed DM.

	Total N = 1030	Women				Men				*p* Value
		Total N = 452 (43.9)	<60 y N = 178 (39.4)	≥60 y N = 274 (60.6)	*p* Value	Total N = 578 (56.1)	<60 y N = 286 (49.5)	≥60 y N = 292 (50.5)	*p* Value	
Age, mean (SD)	62.76 (10.81)	64.56 (11.32)	53.63 (6.13)	71.66 (7.67)	**<0.001**	61.35 (10.18)	53.36 (5.88)	69.19 (6.87)	**<0.001**	**<0.001**
Age at onset, mean (SD)	59.57 (10.70)	61.38 (11.28)	50.67 (6.40)	68.32 (7.79)	**<0.001**	58.15 (10.00)	50.37 (5.88)	65.78 (6.83)	**<0.001**	**<0.001**
Microalbuminuria, %	8.7	6.7	4.0	8.5	0.053	10.5	9.6	11.4	0.480	**<0.01**
ARS, %	11.5	9.1	4.5	12.0	**<0.01**	13.3	10.5	16.1	**0.047**	**0.034**
Hypercholesterolemia, %	64.7	66.9	57.8	72.7	**<0.01**	62.9	56.4	69.3	**<0.01**	0.182
Metabolic Syndrome, %	58.7	61.5	59.3	62.9	0.443	56.6	62.3	51.0	**<0.01**	0.113
HT, %	55.7	57.4	41.4	67.6	**<0.001**	54.5	49.5	59.3	**0.018**	0.352
Hypertriglyceridaemia, %	26.4	22.2	28.3	18.0	**0.011**	29.7	31.6	27.9	0.331	**<0.01**
CVD, %	11.7	6.2	1.7	9.1	**<0.001**	16.1	8.0	24.0	**<0.001**	**<0.001**
Obesity, %	49.4	49.3	50.6	48.5	0.663	49.5	57.0	42.1	**<0.001**	0.949
Sedentarism, %	51.2	58.0	49.4	63.5	**<0.01**	45.8	51.7	40.1	**<0.01**	**<0.01**
Smoking, %	18.5	14.5	17.1	12.9	0.216	21.6	25.6	17.7	**0.021**	**<0.01**
Alcohol, %	47.8	30.5	29.9	30.9	0.821	53.8	48.0	59.4	**<0.01**	**<0.001**
Low adherence Mediterranean Diet, %	7.4	5.4	6.4	4.8	0.470	9.0	12.5	5.6	**<0.01**	**0.027**
Polypharmacy, %	41.4	45.3	31.8	54.0	**<0.001**	38.4	33.6	43.1	**0.019**	**0.026**

SD: standard deviation, ARS: abnormal renal status according to CKD diagnosis in EMRs or current albuminuria. CVD, cardiovascular disease. Low-adherence Mediterranean diet: MEDAS score < 6.

**Table 2:** Therapies for newly diagnosed DM according to associated comorbidities and LDL cholesterol targets achieved.

	Total n/N	Women				Men				*p* Value *
		Total	<60 y	≥60 y	*p* Value	Total	<60 y	≥60 y	*p* Value	
Individuals with HT and Albuminuria in treatment with ACEi/ARBs, n/N’ (%)	44/55 (80.0)	17/21 (81.0)	2/2 (100.0)	15/19 (78.9)	0.471	27/34 (79.4)	8/8 (100)	19/26 (73.1)	0.100	0.890
Individuals with Albuminuria in treatment with ACEi/ARBs n/N’ (%)	54/90 (60.0)	20/30 (66.7)	4/7 (57.1)	16/23 (69.6)	0.542	34/60 (56.7)	15/24 (62.5)	19/36 (52.8)	0.457	0.361
Individuals with ARS in treatment with SGLT2i, n/N’ (%)	50/118 (42.4)	13/41 (31.7)	2/8 (25.0)	11/33 (33.3)	0.650	37/77 (48.1)	17/26 (65.4)	20/51 (39.2)	**0.003**	0.087
Individuals with ARS in treatment with statins, n/N’ (%)	85/118 (72.0)	29/41 (70.7)	3/8 (37.5)	26/33 (78.8)	**0.021**	56/77 (72.7)	16/26 (61.5)	40/51 (78.4)	0.115	0.818
Individuals with ARS with levels of LDLc < 70 mg/dL, n/N’ (%)	35/111 (31.5)	11/40 (27.5)	2/8 (25.0)	9/32 (28.1)	0.859	24/71 (33.8)	6/21 (28.6)	18/50 (36.0)	0.546	0.493
Individuals with ARS with levels of LDLc < 100 mg/dL, n/N’ (%)	69/111 (62.2)	24/40 (60.0)	4/8 (50.0)	20/32 (62.5)	0.519	45/71 (63.4)	12/21 (57.1)	33/50 (66.0)	0.480	0.724

n: patients receiving the referred treatment or achieving the therapeutic goal; N’: number of patients fulfilling the referred condition; HT: hypertension; ARS: Abnormal renal status according to CKD diagnosis in the EMRs or current albuminuria; ACEi/ARBs: angiotensin-converting enzyme inhibitors/angiotensin receptor blockers; SGLT2i: sodium-glucose cotransporter type 2 inhibitors; and LDLc: LDL cholesterol. (*) *p* value between total for women and men.

**Table 3:** Proportion of patients with abnormal renal status (ARS) stratified by social and clinical characteristics.

	ARS % (95% CI)	Crude OR	95% CI	*p* Value
**Sex**				
Men (N = 578)	13.3 (10.5–16.1)	1.54		
Women (N = 452)	9.1 (6.5–11.8)	1		**0.035**
**Age**				
≥70 (N = 253)	20.9 (15.9–25.9)	2.99	1.88–4.76	**<0.001**
60–69 (N = 359)	8.6 (5.7–11.5)	1.07	0.64–1.78	0.801
<60 (N = 418)	8.1 (5.5–10.7)	1		
**Education**				
Elementary (N’ = 365)	14.5 (10.9–18.1)	1.56	1.06–2.29	**0.026**
Superior (N’ = 660)	9.8 (7.5–12.1)	1		
**Social support in chronic disease care**				
None (N’ = 40)	12.5 (2.3–22.8)	1.10	0.42–2.86	0.848
Yes, but not from a familiar (N’ = 40)	10.0 (0.7–19.3)	0.85	0.30–2.45	0.769
Yes, from a familiar (N’ = 947)	11.5 (9.5–13.5)	1		
**Obesity**				
Yes (N = 509)	9.0 (6.5–11.5)	0.62	0.42–0.92	**0.017**
No (N = 521)	13.8 (10.8–16.8)	1		
**HbA1C at 6 months**				
≥8% (N = 79)	12.0 (10.3–19.9)	1.74	0.94–3.21	0.074
<8% (N = 934)	7.3 (8.4–12.6)	1		
**Retinopathy**				
Yes (N’ = 11)	9.1 (0–26.1)	0.76	0.10–6.0	0.795
No (N’ = 1007)	11.6 (9.6–13.6)	1		
**Neuropathy**				
Yes (N’ = 14)	21.4 (0–42.9)	2.11	0.58–7.68	0.257
No (N’ = 1005)	11.4 (9.4–13.4)	1		
**CVD**				
Yes (N = 121)	21.5 (14.2–28.8)	2.43	1.50–3.95	**<0.001**
No (N = 909)	10.1 (8.1–12.1)	1		
**Hypertension**				
Yes (N’ = 568)	14.3 (11.4–17.2)	1.86	1.24–2.81	**<0.01**
No (N’ = 451)	8.2 (5.7–10.7)	1		
**Hypercholesterolemia**				
Yes (N’ = 657)	13.2 (10.6–15.8)	1.62	1.05–2.49	**0.030**
No (N’ = 359)	8.6 (5.7–11.5)	1		
**Psychotic disorders**				
Yes (N’ = 9)	11.1 (0–31.6)	0.95	0.12–7.71	0.966
No (N’ = 1012)	11.6 (9.6–13.6)	1		
**Mood disorders**				
Yes (N’ = 271)	10.0 (6.4–13.6)	0.80	0.51–1.26	0.336
No (N’ = 749)	12.1 (9.8–14.4)	1		
**Cancer**				
Yes (N’ = 77)	10.4 (3.6–17.2)	0.88	0.41–1.88	0.739
No (N’ = 944)	11.7 (9.7–13.8)	1		
**DPP-4 inhibitors**				
Yes (N’ = 147)	16.3 (10.3–22.3)	1.62	0.99–2.64	0.052
No (N’ = 875)	10.7 (8.7–12.8)	1		
**SGLT2 inhibitors**				
Yes (N’ = 256)	19.7 (14.8–24.6)	2.52	1.70–3.78	**<0.001**
No (N’ = 768)	8.9 (6.9–10.9)	1		
**GLP1 analogues**				
Yes (N’ = 86)	11.6 (4.8–18.4)	1.01	0.51–2.01	0.980
No (N’ = 936)	11.5 (9.5–13.5)	1		
**Insulin**				
Yes (N’ = 69)	18.8 (9.6–28.0)	1.87	0.99–3.54	**0.053**
No (N’ = 952)	11.0 (9.0–13.0)	1		
**Antihypertensives therapies ACEi/ARBs**				
Yes (N’ = 531)	14.1 (11.1–17.1)	1.72	1.16–2.56	**<0.01**
No (N’ = 493)	8.7 (6.2–11.2)	1		
**Antihypertensives therapies Non-ACEi/ARBs**				
Yes(N’ = 221)	17.6 (12.6–22.6)	1.96	1.30–2.98	**<0.01**
No (N’ = 803)	9.8 (7.7–11.9)	1		
**Antiplatelet drugs**				
Yes(N’ = 134)	20.1 (13.3–26.9)	2.22	1.38–3.56	**<0.01**
No (N’ = 890)	10.2 (8.2–12.2)	1		
**Corticosteroids**				
Yes (N’ = 30)	13.3 (1.2–25.5)	1.19	0.41–3.46	0.753
No (N’ = 994)	11.5 (9.5–13.5)	1		
**Anticancer medication**				
Yes (N’ = 22)	9.1 (0–21.1)	0.76	0.18–3.31	0.719
No (N’ = 1002)	11.6 (9.6–13.6)	1		
**Immunomodulators**				
Yes (N’ = 17)	5.9 (0–17.1)	0.48	0.06–3.62	0.473
No (N’ = 1007)	11.6 (9.6–13.6)	1		
**Statins**				
Yes (N’ = 655)	13.0 (10.4–15.6)	1.52	0.99–2.32	**0.054**
No (N’ = 369)	8.9 (1.0–11.8)	1		
**Glinides**				
Yes (N’ = 7)	14.3 (0–40.2)	1.29	0.15–10.81	0.814
No (N’ = 1015)	11.5 (9.5–13.5)	1		
**Sulfonylureas**				
Yes (N’ = 38)	13.2 (2.4–24.0)	1.17	0.45–3.06	0.748
No (N’ = 984)	11.5 (9.5–13.5)	1		
**Polypharmacy**				
Yes (N’ = 598)	12.5 (9.9–15.2)	2.78	1.86–4.14	**<0.001**
No (N’ = 423)	10.2 (7.3–13.1)	1		
**Mediterranean diet**				
Low adherence, 0–5 (N’ = 75)	12.0 (4.7–19.4)	0.99	0.47–2.06	0.974
Medium adherence, 6–10 (N’ = 674)	11.9 (9.5–14.3)	0.90	0.41–2.00	0.798
High adherence ≥11 (N’ = 265)	10.9 (7.2–14.7)	1		
**Physical activity**				
Low (N’ = 527)	12.9 (10.1–15.8)	0.77	0.52–1.14	0.188
Moderate or High (N’ = 488)	10.2 (7.5–12.9)	1		

N: total number of patients. N’: number of patients fulfilling the variable referred to. OR: odds ratio. 95% CI: 95% confidence interval. CVD: Cardiovascular diseases. IDPP-4: dipeptidyl peptidase 4 inhibitors. SGLT2 inhibitors: Sodium-glucose cotransporter type 2 inhibitors. GLP1: Glucagon-like peptide-1. ACEi/ARBs: angiotensin-converting enzyme inhibitors/angiotensin receptor blockers.

**Table 4:** Independent variables related to abnormal renal status (ARS) according to multivariate logistic regression.

Variables	OR	95% CI	*p* Value
**Sex**			
Men	1.78	1.16–2.75	0.009
Women	1		
**Age**			
≥70 y	2.96	1.92–4.56	<0.001
<70 y	1		
**Hypertension**			
Yes	1.59	1.03–2.44	0.035
No	1		
**CVD**			
Yes	1.73	1.03–2.90	0.039
No	1		
**HbA1c ≥ 8%**			
Yes	2.26	1.19–4.27	0.01
No	1		

## Data Availability

The data are available upon specific request.
